# Miyoshi Muscular Dystrophy Type 1 with Mutated *DYSF* Gene Misdiagnosed as Becker Muscular Dystrophy: A Case Report and Literature Review

**DOI:** 10.3390/genes14010200

**Published:** 2023-01-12

**Authors:** Joonhong Park, Young Jae Moon, Dal Sik Kim

**Affiliations:** 1 Department of Laboratory Medicine, Jeonbuk National University Medical School and Hospital, Jeonju 54907, Republic of Korea; 2 Research Institute of Clinical Medicine of Jeonbuk National University-Biomedical Research Institute of Jeonbuk National University Hospital, Jeonju 54907, Republic of Korea; 3 Department of Orthopedic Surgery, Jeonbuk National University Hospital, Jeonju 54907, Republic of Korea; 4 Department of Biochemistry and Molecular Biology, Jeonbuk National University Medical School, Jeonju 54907, Republic of Korea

**Keywords:** Dysferlinopathy, Miyoshi muscular dystrophy type 1, *DYSF* gene, c.663 + 1G > C, p.Trp992Arg, targeted panel sequencing

## Abstract

Dysferlinopathy covers a spectrum of muscle disorder categorized by two major phenotypes, namely Miyoshi muscular dystrophy type 1 (MMD1, OMIM #254130) and limb-girdle muscular dystrophy autosomal recessive 2 (LGMDR2, OMIM #253601), and two minor symptoms, including asymptomatic hyperCKemia and distal myopathy with anterior tibial onset (DMAT, OMIM #606768). We report the first Korean MMD1 misdiagnosed as Becker muscular dystrophy (BMD), which was caused by a combination of compound heterozygous c.663 + 1G > C and p.Trp992Arg of the *DYSF* gene. A 70-year-old male previously diagnosed with BMD was admitted for genetic counseling. Since he was clinically suspected to have dysferlinopathy but not BMD, targeted panel sequencing was performed to discover the potential hereditary cause of the suspected muscular dystrophy in the proband. Consequently, two pathogenic single nucleotide variants of the *DYSF* gene, c.663 + 1G > C (rs398123800) and p.Trp992Arg (rs750028300), associated with dysferlinopathy were identified. These variants were previously reported with variant allele frequencies of 0.000455 (c.663 + 1G > C) and 0.000455 (c.2974T > C; p.Trp992Arg) in the Korean population. This report emphasizes the need for common variant screening in the diagnostic algorithms of certain muscle disorders or gene panels with potential pathogenic effects and high rates of recurrent variants.

## 1. Introduction

Dysferlinopathy covers a spectrum of muscle disorders categorized by two major phenotypes, namely Miyoshi muscular dystrophy type 1 (MMD1, OMIM #254130) and limb-girdle muscular dystrophy autosomal recessive 2 (LGMDR2, OMIM #253601), and two minor symptoms, including asymptomatic hyperCKemia and distal myopathy with anterior tibial onset (DMAT, OMIM #606768). Among them, autosomal recessive MMD1 is a skeletal muscle disease featured by onset of distal muscle weakness in early adulthood, which affects the lower and upper limbs except the intrinsic hand muscles. Particularly, muscle atrophy and weakness influence the soleus and gastrocnemius muscles, which spread to the gluteal and thigh muscles later. Affected individuals presented with difficulty walking, difficulty in climbing stairs, and impaired tiptoe standing but usually remained ambulatory. Furthermore, muscle biopsies show myopathic as well as dystrophic changes with necrosis and serum creatine kinase (CK) are elevated [1]. In this context, the development of next-generation sequencing (NGS) is an efficient method of arriving at a genetic diagnosis in a varied population naive to molecular testing, thereby enabling simultaneous sequencing of thousands of genes more rapidly and at cheaper costs than traditional Sanger sequencing. Moreover, NGS-based genetic testing may be relevant in resource-poor settings, where radiological and biochemical facilities may not be available readily or are expensive prohibitively [2]. Because targeted NGS or gene panel sequencing is designed to reveal the causal variants for genes known to be related to specific rare diseases, the uniform and ultradeep coverage allows high sensitivity as well as specific variant calling for rare genetic variants [3]. Furthermore, gene panel sequencing has been performed successfully to inherited diseases with causal genes involved in the common disease-related pathways and with genetic heterogeneity, including overlapping phenotypes, locus heterogeneity, and allelic heterogeneity [4,5].

To date, several genetic studies of dysferlinopathies diagnosed as MMD1 or LGMD2B have been reported in Koreans [6,7,8,9,10,11,12]. Here, we report the first Korean MMD1 misdiagnosed as Becker muscular dystrophy (BMD), which was caused by a combination of compound heterozygous c.663 + 1G > C and p.Trp992Arg of the *DYSF* gene.

## 2. Materials and Methods

### 2.1. Multiplex Ligation-Dependent Probe Amplification

**Figure 1:**
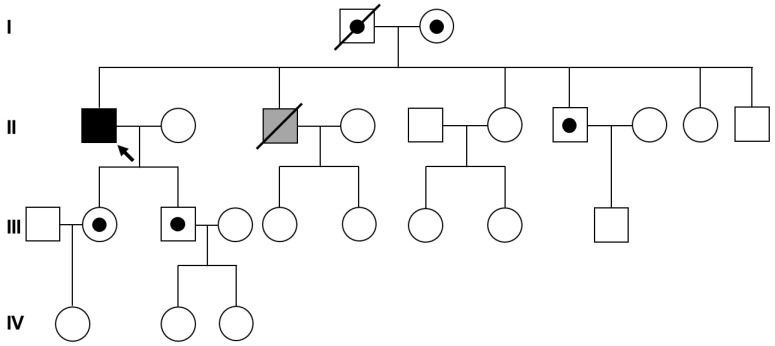
Pedigree analysis. The pedigrees of the proband (arrow) with Becker muscular dystrophy and his family members. The unaffected parents of the proband are obligate heterozygotes. The gray symbol indicates that the family member was clinically suspected to have dysferlinopathy but not confirmed genetically.

To detect duplications or deletions of the *DMD* gene in the proband (II-1 in Figure 1) and his daughter (III-2 in Figure 1), multiplex ligation-dependent probe amplification (MLPA) was conducted using a SALSA^®^ MLPA^®^ Probemix P035 DMD-1/2 (MRC Holland, Amsterdam, The Netherlands) according to manufacturer protocols. Capillary electrophoresis and fragment analysis were performed using a 3500xL Dx Genetic Analyzer (Applied Biosystems, Carlsbad, CA, USA). Comparative copy number variation data were estimated using Coffalyser.Net software ver. 210604.1451 (MRC Holland) according to manufacturer instructions. The generated peak signals were normalized to the manufacturer control probes and those of gender-matched normal DNA as reference. Estimating that the probe normally targets two copies, a probe to peak ratio of 0.7–1.3 was considered as a normal copy number (wild type). Further, a probe to peak ratio of 1.3–1.6 was considered to indicate a heterozygous duplication (gain of one copy number), and a probe to peak ratio of 0.4–0.7 was assumed to indicate a heterozygous deletion (loss of one copy number).

### 2.2. Targeted Panel Sequencing

Targeted panel sequencing using a custom hereditary muscular dystrophy panel developed by Green Cross Genome (Yongin, Korea) (Table S1; Supplementary Material) was conducted in the proband (II-1 in Figure 1). Paired end (PE) massively parallel sequencing was performed using a NextSeq500 instrument (Illumina, San Diego, CA, USA) with a 300 PE cycles (150 × 2) and high output flow cell to identify the variant given the causation of a hereditary myopathy. The sequencing reads were mapped with the human genome reference assembly GRCh38 (hg38) using the Burrows–Wheeler aligner (BWA). Base calling, alignment, variant calling, annotation, and quality control reporting were sequentially processed based on germline short variant discovery proposed by the Genome Analysis Tool Kit best-practice pipeline workflow (https://gatk.broadinstitute.org/hc/en-us/, accessed on 8 July 2020). As a result, a yield on a target of 313,188,660 reads was generated from the proband’s sample via estimation of the sequence quality along all mapped sequences. The mean coverage of the read depth (×) was 270, and the percentage of target bases > 30× was 99.9%. The pathogenic effect of the sequence variants was carefully interpreted by medical laboratory physician based on the standards and guidelines of the Joint Consensus Recommendation of the American College of Medical Genetics and Genomics (ACMG) and Association for Molecular Pathology (AMP) for classifying the pathogenic variants [13]. Briefly, the filtering scheme applied to discover the potential harmful variant is as follows: (1) variants located near or within the exons of protein-coding genes; (2) allele frequencies of variants less than 0.1%; (3) variants causing nonsynonymous or nonsense changes in codons within exons, changing the highly conserved splice sites, or leading to frameshift mutations; (4) de novo variants or compound heterozygous or homozygous variants of the same gene identified in the proband only. The certain muscle disease is most likely regarded to be sporadic or autosomal/X-linked recessive inheritance because the proband’s parents were not affected. In addition, the pathogenic effects of filtered variants were evaluated using ClinVar (https://www.ncbi.nlm.nih.gov/clinvar/, accessed on 3 August 2020). The allele frequencies of the filtered variants were estimated in the general population using the genome aggregation database (gnomAD, https://gnomad.broadinstitute.org/, accessed on 3 August 2020) and in the Korean ethnic population using the Korean Reference Genome Database (KRGDB, http://152.99.75.168/KRGDB/, accessed on 3 August 2020).

## 3. Results

A 70-year-old male (II-1 in Figure 1) visited the outpatient department of orthopedic surgery of Jeonbuk National University Hospital (Jeonju, Korea) for genetic counseling. He had previously been diagnosed with BMD 25 years ago through clinical findings and a muscle biopsy in Samsung Jeil Hospital (Seoul, Korea). A muscle biopsy was performed in the lateral rectus femoris, and pathological findings showed variation in fiber size, inflammatory infiltration, and degeneration and regeneration of muscle fiber without vacuoles. However, no additional immunohistochemistry or Western blotting was performed on the muscle biopsy (data not shown). The patient was normal at birth and healthy until the age of 16 years; at about 18 years of age, he started having trouble climbing stairs because of weakness in the dorsiflexion of his ankle. After several years, he also had difficulty squatting by Achilles tendon contracture and showed a waddling gait. At 45 years of age, his lower extremity muscle weakness aggravated such that he had to use a wheelchair for ambulation. The muscle weakness of the upper extremities progressed slowly compared to that of the lower extremities. He had difficulty elevating the shoulders in his 50s and hard flexion of the elbow in his 60s. In his familial history, his younger brother also had muscle weakness, but his other siblings, parents, and children were all healthy. There was no specific history other than musculoskeletal weakness.

In the physical examination, muscle power was lower in both the lower and upper extremities. The muscles of the hip, knee, ankle, shoulder, and elbow were observed to be grade 1 in the manual muscular strength test (MMT); the wrist flexors and extensors were MMT grade 2; and the flexors and extensors of the fingers were MMT grade 3. All joints showed no contracture and full passive range of motion (ROM). His sensations were intact, and the deep tendon reflex showed hypoactivity without a pathologic reflex. Laboratory tests showed an elevated serum CK level of 352 IU/L, but there were no other abnormal findings (Table 1). We, therefore, suspected that the proband had MMD1 among the dysferlinopathy types based on the following clinical symptoms. The first recognized symptoms were at the distal part of the leg, which gradually progressed to difficulty with climbing stairs and walking. In addition, the onset of muscle weakness in the upper extremities appeared relatively later than that in the legs, and the proband still has small muscle strength of the hands. Because the proband’s parents (I-1 and I-2 in Figure 1), children (III-2 and III-3 in Figure 1), and grandchildren (IV-1, -2, and -3 in Figure 1) showed no clinical manifestations related to muscular dystrophy, even though his younger brother (II-3 in Figure 1) only presented with similar phenotypes, segregation analysis and genetic counseling were additionally conducted to resolve the genetic origin of dysferlinopathy in the proband.

**Figure 2:**
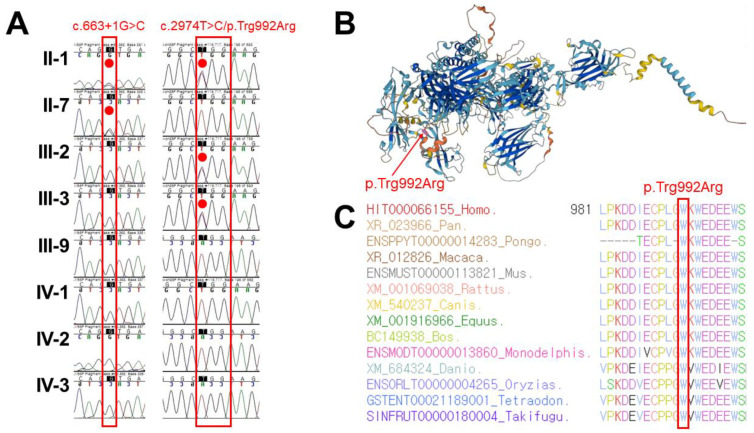
Sanger sequencing, protein structure prediction, and conservation comparison of the p.Trp992Arg variant of the *DYSF* gene. (**A**) Sanger sequencing confirmation of occurrence of the compound heterozygous *DYSF* variants c.663 + 1G > C and c.2974T > C; p.Trp992Arg as a consequence of autosomal recessive inheritance in the proband (II-1), which is highlighted with the red dot. (**B**) Protein structure study using AlphaFold shows very high per-residue confidence metric called predicted local distance difference test (pLDDT) of 83.37 for p.Trp992Arg of the *DYSF* gene. The protein residue of p.Trp992 is highlighted in pink and indicated by a red arrow. (**C**) Sequence alignments of the DYSF protein in several vertebrate species. The protein sequence of the Trp992 residue is conserved highly across the compared species between Human (HIT000066155_Homo) and Takifugu (SINFRUT00000180004_Takifugu), which is highlighted with the red box.

After genetic analysis, no duplications or deletions were identified in the *DMD* gene in the genomic DNA isolated from the proband and his daughter. Thus, previous diagnosis of BMD was excluded. Consequently, targeted panel sequencing identified two pathogenic single-nucleotide variants of the *DYSF* gene (Reference transcript ID of *DYSF*: NM_003494.3) associated with dysferlinopathy, namely c.663 + 1G > C (rs398123800) and p.Trp992Arg (rs750028300), which were considered as the best candidates for the autosomal recessive muscular dystrophy. These variants were previously reported with variant allele frequencies of 0.000455 for c.663 + 1G > C [8,9,10,11] and 0.000455 for c.2974T > C; p.Trp992Arg [7,9,10] in the Korean population. Sanger sequencing was additionally performed to determine the carrier status for the family members, and the genetic origin of these *DYSF* variants were confirmed to be autosomal recessive (Figure 2A).

DYSF protein structure was studied to examine amino acid structure stability for the human proteome, using AlphaFold, a protein structure database which predict a protein’s 3D structure from its normal protein sequence [14]. As a result, protein structure study showed a per-residue confidence metric called predicted local distance difference test (pLDDT) of 83.37 for p.Trp992Arg of the *DYSF* gene (Confident, 70 < pLDDT < 90) (Figure 2B). In addition, sequence alignment of the DYSF protein in multiple vertebrate species was compared, and protein sequence of the Trp992 residue is conserved highly across the compared vertebrate species (Figure 2C). Based on these genetic results and clinical manifestations, compound heterozygous c.663 + 1G > C and p.Trp992Arg of the *DYSF* seems to be most likely candidate for the genetic cause responsible for clinical manifestations in the proband with autosomal recessive dysferlinopathy diagnosed as MMD1. 

## 4. Discussion

Since the dystrophinopathies comprise a set of X-linked muscle disorders related to causative variants in the *DMD* gene ranging from mild to severe, covering BMD, Duchenne muscular dystrophy (DMD), and DMD-related dilated cardiomyopathy [15], differential diagnosis is essentially required. In particular, BMD is characterized by late-onset skeletal muscle weakness. Individuals who presented with calf muscle hypertrophy were misdiagnosed previously to have BMD; a slowly progressing course of muscle weakness was common in all these individuals [16]. Among the dysferlinopathy phenotypes, MMD1 is featured by a distinct elevation in CK level compared to other distal myopathies; here, the serum CK concentration ranges from 20 to 150 times the normal level. Indeed, increased CK concentrations are often observed prior to clinical manifestations or symptoms in such individuals. Electromyography shows brief and small myopathic motor-unit potentials as well as early recruitment. Very atrophic and weak gastrocnemius muscles reduced recruitment and may demonstrate long-duration polyphasic motor-unit potentials [17]. Muscle biopsy may demonstrate “end-stage” disease with loss of most muscle fibers, fatty replacement, and widespread fibrosis in the severely defected gastrocnemius muscle. About 10% of affected individuals with dysferlinopathy show an infiltration of inflammatory cells in the muscle biopsies. Dysferlin is absent in the plasma membrane, whereas scattered granular staining may be observed in the nuclear membrane or cytoplasm by muscle biopsy in MMD1 [18]. However, some individuals may also present with only a marked elevation of the serum CK level. This condition is regarded usually as a presymptomatic presentation of myopathy in an individual who eventually develops weakness and muscle atrophy. Sometimes the calf muscles are enlarged, and this finding may be misdiagnosed as a dystrophinopathy such as BMD or DMD. In the present case, our proband was previously misdiagnosed to have BMD in presymptomatic hyperCKemia through muscle biopsy. Muscle biopsies may show nonspecific dystrophic features of varying degrees with endomysial connective tissue proliferation, muscle fiber necrosis, and fiber size variability. Inflammatory infiltrates, which are mainly perivascular, have also been observed in several cases, as described previously [16].

Dysferlinopathy usually presents with early adult onset and elevated serum CK concentrations. However, the pathological findings on routine histopathological and immunohistochemical stains and heterogeneity of clinical manifestations make it difficult to diagnose dysferlinopathy [19]. Several patients with dysferlinopathy have been previously misdiagnosed with inflammatory myopathy [19,20] and juvenile polymyositis [21,22]. In the case of dysferlinopathy, most patients received muscle biopsies for increased inflammatory responses [23,24], even those who were less affected clinically, suggesting that this sign is a relatively early feature [25]. Thus, dysferlinopathy can be potentially misdiagnosed as inflammatory myopathy for several reasons. In muscle specimens of inflammatory myopathy as well as dysferlinopathy, inflammatory cell infiltration is observed frequently. Many affected individuals with dysferlinopathy have shown normal muscle strength prior to onset of sign, which is suggestive of an acquired myopathy [26]. Inflammatory cell infiltration may be related to prolonged release of endogenous molecules and impaired secretion of cytokines, including adenosine triphosphates, heat shock proteins, and high mobility group box 1, because of compromised membrane repair [27]. Therefore, affected individuals with dysferlinopathy are most vulnerable to misdiagnosis as polymyositis. Specifically, misdiagnosis of LGMDR2 as polymyositis is a common occurrence. These two diseases have some common clinical features, including increased CK levels, asymmetric weakness of the limbs, and electromyographic alterations indicating muscle-derived injury [21]. Thus, molecular analysis of the genes associated with dysferlinopathy or estimation of the presence of dysferlin protein is a very useful approach for distinguishing dysferlinopathy from other muscular disorders.

As of November 2022, a total of 49 different disease-causing *DYSF* variants have been reported in 72 Korean persons diagnosed with MMD1 or LGMDR2 (Table 2) [6,7,8,9,10,11,12], which can be categorized under five types of variants: 20 missense, 10 nonsense, 10 frameshift, 7 splicing site, and 2 silent variants. This distribution of mutation types reflects the genetic variations across the Korean population and wide spectrum of *DYSF* variants; however, there is a lack of locational hotspots, which emphasizes the requirement for comprehensive molecular study of the very large *DYSF* gene. Of the reported variants, c.2494C > T/p.Gln832*, c.1284 + 2T > C, and c.663 + 1G > C are the three most common disease-causing *DYSF* variants. The domain architecture of dysferlin is predicted to comprise seven C2 domains (C2A to C2G), three Fer domains (FerA, FerB and FerI), two DysF domains with one nested within the other, and a C terminal transmembrane domain. C2 domains are observed in hundreds of proteins, and many are known to link to phospholipids or proteins, often in a calcium-dependent manner [28]. On the other hand, dysferlinopathy leading to mutations are scattered throughout the length of the protein, however many located in the DysF domains [29]. One of these DysF domains is inserted into the domain of the other by gene duplication, forming a two-part (N and C terminals) outer DysF domain and an inner DysF domain [30]. It is notable that the p.Trp992Arg variant, together with other variants such as the p.Trp999Arg/Cys, p.Ser1000Phe, p.Arg1040Trp, and p.Arg1046His representing about 71% of the 160 missense variants, were located in the IdysF domain in the Japanese population [15]. Although this accumulation in the IdysF domain may be less attributable to the lower variant allele frequency 0.000455 of c.2974T > C in KRGDB, the accumulation of other missense variants in the vicinity indicates the likely pivotal function of this domain. Although the functions of seven cytosolic C2 domains with calcium-binding activities have been clarified [31], the functions of the Fer and DysF domains remain essentially unknown. The DysF domains are composed of unusual internal duplications, where the IdysF domain is inserted between the N- and C-terminal parts of a homologous domain with a unique fold held together by the stacking of arginine and tryptophan residues [32].

## 5. Conclusions

In this report, we present the genetic diagnosis of a compound heterozygosity in the *DYSF* gene as a cause of MMD1 misdiagnosed as BMD by muscle biopsy in a Korean family. This report emphasizes the need for common variant screening in the diagnostic algorithms of certain muscle disorders or gene panels with potential pathogenic effects and high rates of recurrent variants.

## Figures and Tables

**Table 1:** The patient’s clinical and laboratory findings.

Parameters	Results	Normal Range	Parameters	Results	Normal Range
BMI (kg/m^2^)	25.7	18.5–25	Albumin (g/dL)	4.6	3.5–5.2
WBC (×10^3^/μL)	7.29	4.0–10.0	Total Bilirubin (mg/dL)	0.89	0–1.2
RBC (×10^6^/μL)	5.02	4.2–6.3	ALP (IU/L)	117	35–130
Hemoglobin (g/dL)	15.5	13.0–17.0	AST (IU/L)	32	0–37
Platelet (×10^3^/μL)	197	130–400	ALT (IU/L)	26	0–41
BUN (mg/dL)	9.8	6–20	γ–GT (IU/L)	43	6–71
Creatinine (mg/dL)	0.18	0.6–1.2	LDH (IU/L)	203	135–225
Ca (mg/dL)	9.4	8.6–10.2	Creatine kinase (IU/L)	352	50–250
P (mg/dL)	2.8	2.7–4.5	Triglyceride (mg/dL)	127	0–200
Uric acid (mg/dL)	5.6	3.4–7.0	Cholesterol (mg/dL)	115	120–220
Glucose (mg/dL)	109	70–110	HDL (mg/dL)	41	35–75
HbA1c (%)	5.1	4.2–5.9	LDL (mg/dL)	62	0–130

**Table 2:** 49 reported *DYSF* variants in 72 Korean Miyoshi muscular dystrophy type 1 or limb-girdle muscular dystrophy autosomal recessive type 2.

Base Change	Codon Change	Reported VAF	rsID	gnomAD	KRGDB	References
c.75del	p.Ala26Argfs * 6	1	N/A	0	0	[9]
c.313dup	p.Leu105Profs * 43	1	N/A	0	0	[9]
c.610C > T	p.Arg204 *	1	rs373585652	0.000007953		[9]
c.663 + 1G > C	Splicing error	19	rs398123800	0.000003978	0.000455	[8,9,10,11]
c.675G > T	p.Gln225His	1	N/A	0	0	[8]
c.757C > T	p.Arg253Trp	1	rs149827237	0.0001233		[8]
c.823del	p.Glu276Serfs * 12	2	N/A	0	0	[8,11]
c.845T > C	p.Ile282Thr	3	N/A	0	0	[8,9]
c.895G > A	p.Gly299Arg	1	rs121908963	0.00003186 *		[9]
c.937 + 1G > A	Splicing error	5	rs201869739	0.00003579		[9,10,11]
c.938-1G > A	Splicing error	1	N/A	0	0	[9]
c.1053T > G	p.(=)	1	rs199955501	0.0002864		[8]
c.1129C > T	p.Arg377 *	2	rs758180890	0.00002387		[9,10]
c.1165G > C	p.Glu389Gln	3	N/A	0	0	[6,8]
c.1284 + 2T > C	Splicing error	20	rs398123765	0.00001596	0	[8,9,10,11]
c.1464del	p.Gly489Glufs *4	5	N/A	0	0	[8,9,10]
c.1579G > T	p.Gly527Cys	1	N/A	0	0	[8]
c.1646del	p.Gly549Valfs * 78	1	N/A	0	0	[11]
c.1663C > T	p.Arg555Trp	1	rs377735262	0.00002916	0	[12]
c.1665G > C	p.(=)	1	N/A	0	0	[11]
c.2248C > T	p.Gln750 *	1	N/A	0	0	[8]
c.2494C > T	p.Gln832 *	28	rs199543257	0.00001988	0.000455	[7,8,9,10,11]
c.2964C > A	p.Cys988 *	1	N/A	0	0	[8]
c.2974T > C	p.Trp992Arg	3	rs750028300	0.000003976	0.000455	[7,9,10]
c.2997G > T	p.Trp999Cys	11	rs28937581	0.00001193	0.001818	[7,8,9,10,11] [12]
c.3032-1G > A	Splicing error	2	N/A	0	0	[8]
c.3102C > G	p.Tyr1034 *	1	N/A	0	0	[9]
c.3113G > A	p.Arg1038Gln	2	rs150877497	0.00005186		[8,11]
c.3275G > A	p.Arg1092His	2	rs758284713	0.00009168		[8]
c.3276_3281dup	p.Arg1093_Trp1094insCysArg	1	rs758284713	0.000003986		[9]
c.3289C > T	p.Arg1097Cys	1	rs147483765	0.0003622		[8]
c.3307A > T	p.Lys1103 *	1	N/A	0	0	[7]
c.3407del	p.Gly1136Valfs * 2	2	rs778088008	0.000003976		[8,9]
c.3875_3880del	p.Val1292_Gln1293del	1	N/A	0	0	[9]
c.4200dup	p.Ile1401Hisfs * 8	1	N/A	0	0	[7]
c.4434G > A	p.Trp1478 *	1	rs766016391	0.000004045		[8]
c.4742G > A	p.Arg1581His	1	rs185596534	0.0005567		[8]
c.4795-2A > G	Splicing error	1	N/A	0	0	[8]
c.5078G > A	p.Arg1693Gln	2	rs779987458	0.000008007		[8,10]
c.5090G > C	p.Arg1697Pro	1	rs777701226	0.00002806		[8]
c.5095T > C	p.Ser1699Pro	1	N/A	0	0	[8]
c.5287G > A	p.Glu1763Lys	1	N/A	0	0	[10]
c.5607dup	p.Arg1870Glufs * 12	2	N/A	0	0	[8,9]
c.5776C > T	p.Gln1926 *	1	N/A	0	0	[9]
c.5804C > T	p.Pro1935Leu	1	rs1254719758	0.00003185		[10]
c.5911T > C	p.Cys1971Arg	1	N/A	0	0	[8]
c.5939T > A	p.Ile1980Lys	1	N/A	0	0	[9]
c.6057-2A > C	Splicing error	2	N/A	0	0	[11]
c.6096C > G	p.Tyr2032 *	1	rs1012723902	0.000003976		[8]

Reference nucleotide ID of DYSF: NM_003494.4; * gnomAD_Genomes v.2.1.1. rsID, Reference SNP cluster ID; VAF, variant allele frequency; gnomAD, The Genome Aggregation Database v.2.1.1 exomes; KRGDB, Korean Reference Genome DB (1100 individuals: The 2nd phase); N/A, not available

## Data Availability

Not applicable.

## Supplementary Materials

The following are available online at https://www.mdpi.com/article/10.3390/genes14010200/s1, Table S1: A list of 68 selected genes in a custom hereditary muscular dystrophy panel.
