# Supplementary material for: Miyoshi Muscular Dystrophy Type 1 with Mutated DYSF Gene Misdiagnosed as Becker Muscular Dystrophy: A Case Report and Literature Review
Source: Genes (Basel). 2023 Jan 12;14(1):200. doi: 10.3390/genes14010200 (PMC9859596; doi:10.3390/genes14010200)
Supplement: Supplementary file 1 [file genes-14-00200-s001.zip › genes-2075883-supplementary.pdf]

Supplementary Table S1. A list of 68 selected genes in a custom hereditary muscular dystrophy panel

ACTA1	*ANO5*	*ATP2A1*	*BAG3*	*BIN1*	*CAPN3*	*CAV3*
CCDC78	*CFL2*	*CLCN1*	*CNTN1*	*COL6A1*	*COL6A2*	*COL6A3*
CRYAB	*DAG1*	*DES*	*DMD*	*DNAJB6*	*DOK7*	*DYNC1H1*
DYSF	*EMD*	*FHL1*	*FLNC*	*FKRP*	*FKTN*	*GAA*
GNE	*IGHMBP2*	*ISPD*	*ITGA7*	*KBTBD13*	*KLHL40*	*KLHL41*
LAMA2	*LARGE*	*LDB3*	*LMNA*	*MTM1*	*MYH7*	*MYOT*
NEB	*PABPN1*	*PLEC*	*POLG*	*POMGNT1*	*POMT1*	*POMT2*
RYR1	*SEPN1*	*SGCA*	*SGCB*	*SGCD*	*SGCE*	*SGCG*
SYNE1	*SYNE2*	*TCAP*	*TNNT1*	*TPM2*	*TPM3*	*TRIM32*
TRPV4	*TTN*	*VCP*	*VPS13A*	*VRK1*		
